# Investigating the Welfare of Zoo‐Housed *Cryptoprocta ferox*: Behavioral Observations and Hormonal Profiling

**DOI:** 10.1002/zoo.21884

**Published:** 2024-12-19

**Authors:** Giovanna Marliani, Silvia Calamandrei, Giovanni Buonaiuto, Pier Attilio Accorsi, Camillo Sandri, Caterina Spiezio

**Affiliations:** ^1^ Department of Veterinary Medical Science Alma Mater Studiorum – University of Bologna Bologna Italy; ^2^ Parco Natura Viva – Garda Zoological Park Bussolengo Italy

**Keywords:** animal behavior, cortisol, endocrinology, fossa, welfare

## Abstract

The global decline of species necessitates intensive conservation efforts, including ex‐situ breeding programs to safeguard endangered populations. However, managing welfare and reproduction in zoological gardens can present several challenges. This study aims to explore behaviors and endocrinological responses of two specimens of fossa (*Cryptoprocta ferox*) under human care. One hundred and twenty hours of observation through focal animal sampling continuous recording and 67 fecal samples were collected. From fecal samples, cortisol (FCM), progesterone (FPM), and testosterone (FTM) metabolites' concentrations were analyzed using Radio Immuno Assay. According to our results, higher FTM (*p* < 0.05) and FPM levels at the beginning of the study, showing a potential alignment with the breeding season. When the male entered the female outdoor area, he recorded longer durations of seeking and marking behaviors (*p* ≤ 0.01), along with lower FCM levels (*p* < 0.05), suggesting an enriching effect. In the same period, the female's FCM level was higher (*p* ≤ 0.01), indicating potential stress. When both animals were in their respective outdoor areas, the FCM level of the female remained significantly higher (*p* < 0.05), and the male showed a peak in pacing duration (*p* < 0.01), implying a potential stress or frustration status. In her indoor area, the female exhibited significantly shorter durations of locomotion, marking, and seeking (*p* < 0.01) and did not show any abnormal behavior, likely due to the reduced complexity of the environment. However, we cannot exclude that her complete visual and olfactory separation from the male and visitors may also have contributed to a decrease in her overall stress levels. The findings highlight the interplay between hormonal fluctuations and behavioral responses in the two zoo‐housed fossa, providing essential insights for fostering their well‐being and facilitating conservation efforts.

## Summary

- Complexity of environment is necessary to promote physiological behaviors in fossa.
- Socialization can be a stressful process for fossa.
- Fecal steroid hormones help monitor welfare and reproductive status in fossa.

## Graphical abstract

This study examines the interplay between hormonal fluctuations and behavioral responses in two zoo‐housed fossa, offering essential insights to enhance their well‐being and support conservation efforts.

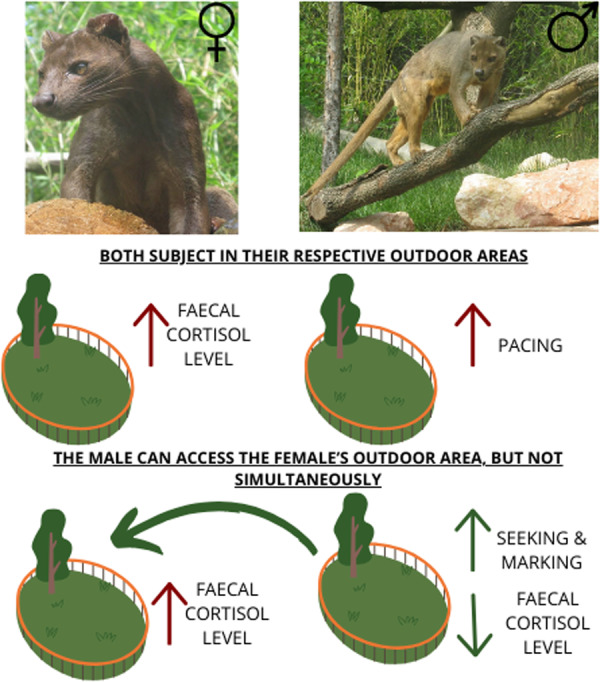

## 1 Introduction

The decline of numerous species worldwide has necessitated extensive conservation efforts, prompting the implementation of various strategies to protect endangered populations (e.g., Brichieri‐Colombi et al. 2019; Martin‐Wintle et al. 2019). Among these approaches, ex‐situ management, including breeding programs, has played a crucial role in rescuing several species from the brink of extinction (Roe, Frank, and Kingsbury 2015). However, effectively managing welfare and reproduction in a controlled environment poses significant challenges, particularly when trying to replicate the intricate environmental conditions that endangered species demand, including habitat size, complexity, and arrangement of relevant stimuli (Hosey, Melfi, and Pankhurst 2013; Canessa et al. 2016; McGowan, Traylor‐Holzer, and Leus 2017).

Ensuring high levels of animal welfare is both a fundamental ethical and legal responsibility (El‐Sabrout et al. 2024). This urgency extends not only to farm animals but also encompasses animals in zoos (Krebs et al. 2018). Moreover, ensuring the highest possible standards of animal welfare is not merely an obligation but also an essential prerequisite for zoos to achieve their educational and conservation objectives and accreditations (Wolfensohn et al. 2018). However, developing welfare strategies for any species poses challenges, as it requires careful consideration of numerous factors, including their complete range of physiological and behavioral requirements (Clubb and Mason 2007). In this context, research plays a crucial role in understanding the distinctive characteristics of species in the wild, such as their ecology, social organization, and ethology. Findings from such research are essential for identifying key points to provide appropriate well‐being in a controlled environment (Wolfensohn et al. 2018).

Consequently, it is vital to monitor and assess the well‐being of each individual using suitable methodologies (Smith et al. 2023). Several techniques can be used to measure animal welfare in zoo animals. However, the most common methods involve monitoring physiological indicators (e.g., hypothalamic–pituitary–adrenal axis activation) and behavioral indicators (e.g., stereotypies or unusual activity levels; Hill and Broom 2009; Hosey, Melfi, and Pankhurst 2013; Whitham and Wielebnowski 2013). Measuring and documenting behavior is crucial for evaluating zoo animal welfare. Results of field studies that investigate behaviors and activity patterns of wild animals (Di Bitetti, Paviolo, and De Angelo 2006; Ridout and Linkie 2009; Rozhnov et al. 2011; Lynam et al. 2013) can serve as a baseline for knowing the ethology of a species. However, it is important to underline that the presence or absence of natural behaviors is not necessarily indicative of, respectively, positive welfare or suffering status. Indeed, in considering positive welfare, it is more important to monitor the expression of highly motivated and highly rewarded behaviors, which can be either natural or unnatural (only exhibited in captivity; Learmonth 2019). Usually, behavioral monitoring is the preferred method to monitor animal welfare, because it is cost‐effective, noninvasive, and can be regularly and easily recorded by trained caretakers. Indeed, caretakers in zoological institutions usually have extensive knowledge of their animals' behavior, allowing them to detect subtle changes that an external observer might miss (Wemelsfelder et al. 2001; Wemelsfelder 2007). In zoos, behavioral analysis has been effectively used as a method to monitor welfare (Biolatti et al. 2016; Wagman et al.^2018^). Given the relatively low output of research on the fossa species and its management in a controlled environment, conducting ethological surveys may serve as a valuable step toward the improvement of best‐practice guidelines for its conservation and ex‐situ management (Harley, O'Hara, and Rose 2021).

Fossa (*Cryptoprocta ferox*) is the largest Madagascar carnivore species belonging to the family Eupleridae, which is one of the most endangered and least researched groups of carnivores globally (Brooke et al. 2014; Farris et al. 2015; Merson, Dollar, Tan, et al. 2019). Its weight averages from 6.1 kg in females to 7.4 kg in males, and its length can range from 61 to 80 cm (Dollar 2006; Hunt 2022). Out of the breeding season, both males and females are typically solitary, but some males may aggregate and form coalitions of two or three individuals. They have both arboreal and terrestrial habits, and their home range extends from 12 to 89 km^2^. The home range of males can overlap with those of conspecifics of both sexes, whereas females usually share territory only with related females (Wyza et al. 2020; Lührs and Kappeler 2013). They live in rainforests and deciduous forests, from the coastal plains to the mountainous areas at 2500 m altitude. The fossa's population density varies from 0.17 to 0.22 individuals/km^2^ (Hawkins 2003; Hawkins and Racey 2005; Gerber et al. 2010). Their significance lies in being potential keystone species, given their role in preying on lemurs and other small and medium‐sized animals, such as rodents, birds, and wild pigs. They are ambush hunters and use their retractable claws to prey (Hawkins and Racey 2008; Hunt 2022). According to the IUCN Red List, they are classified as a Vulnerable species, and their population is decreasing (Hawkins 2016). However, considering Madagascar's Classification for Animal species (Decree No. 2006‐400 of June 13, 2006, on the classification of wildlife species), fossa is listed as Category 1 Class 2, and, therefore, can be hunted for authorized commercial, sport, or regulatory purposes (Keane et al. 2011; Merson, Dollar, Johnson, et al. 2019).

The fossa has been kept in ex‐situ environments since the 19th century (Hornsey 1999). However, their unique ethology and ecology present substantial challenges to successful management in zoos. In particular, their solitary nature and need for extensive space complicate their management, making these animals prone to developing abnormal and stereotypic behaviors, especially pacing, in controlled environments (Harley, O'Hara, and Rose 2021). Key strategies to mitigate these issues can include housing dimensions, particularly when fossa are kept with conspecifics or in adjacent enclosure; an adequate enrichment program and structures (e.g., dense cover enclosures that provide hiding spots and vertical space); restricted views of visitors and other species; and variability in the feeding schedule (Harley, O'Hara, and Rose 2021). Considering the challenges and opportunities posed by ex‐situ conservation programs, our research aimed to assess the behavior of two specimens of fossa under human care: a resident male and a new‐arrival female. The animals were housed in two separate enclosures, and the study was conducted during the initial period following the female's arrival, during which she was familiarizing herself with the new enclosure and the presence of the male in the adjacent enclosure (auditory and olfactory). Employing a comprehensive approach, we examined both ethological and endocrinological parameters, encompassing behavioral observations and hormone analysis, to gain deeper insights into the species‐specific behaviors, stress levels, and reproductive status. Additionally, our findings may contribute to a broader understanding of managing endangered species under human care, enriching the existing knowledge.

## 2 Materials and Methods

### 2.1 Subjects and Environment

The current study was carried out on two specimens of Madagascar fossa (*Cryptoprocta ferox*) housed at Parco Natura Viva (Bussolengo, Verona, Italy). Both subjects were 14 years old, but they arrived at the zoological garden at different times: the male in March 2009 and the female in March 2015. Upon the male's arrival, he was already experiencing stereotypical locomotor repetitive behavior (pacing), which was partially fixed through an intensive 6‐8 months enrichment program (Grisa et al. 2013). Additionally, he had difficulties to locate food in the enrichment tools using his sense of smell; this issue was also addressed with an appropriate enrichment program (Grisa et al. 2013).

Due to the territoriality of this species, the two animals were housed in two different adjacent enclosures, with an outdoor and indoor enclosure area each. The two enclosures were separated by a neutral area with vegetation, and the outdoor enclosure was connected by two tunnels with two sliding “guillotine” doors, each always closed. The outdoor enclosures were delimited by a fence made of wooden logs and a net and were covered with a high net, which allowed the development of vegetation on which animals climb. The areas were equipped with vegetation, trees, ropes, and wooden furnishings to allow arboreal locomotion, as well as caves in which the fossa could rest. Both the outdoors had a window from which visitors could observe the animals. The indoor enclosure was not visible to the public, and it included a wooden house with wooden furnishings. The animals were always physically separated but could perceive each other's presence via olfactory and visual observations. When one of the two animals was in their indoor area, the tunnels connecting the two outdoor enclosures were opened to allow the fossa to explore the outdoors of the animal kept indoor. Both fossa were provided with an enrichment program, water ad libitum, and a one daily meal in the indoor area.

### 2.2 Behavioral Data Collection

In the initial 14 days, the observer conducted preliminary observations to create an ethogram (reported in Table S1), which was later supplemented with information from existing literature. This period also allowed the fossa to become accustomed to the observer's presence. For the entire study, the observer was the same (S.C.).

The experimental phase, which lasted from May 27, 2015, to July 13, 2015, was divided into five different study periods (A, B, C, D, and E; Table 1). During period A (May 27, 2015–June 5, 2015), the male was in his outdoor enclosure during the day (9 a.m.–6 p.m.), while the female remained in the indoor enclosure. During the night (6 p.m.–9 a.m. of the following day), the male was in the indoor enclosure while the female was allowed to go to her outdoor enclosure. In the second period, B (June 8, 2015–June 16, 2015), the alternation of the individuals in their respective enclosures was the same as in the first period. However, in the afternoon, from 3 to 4 p.m., the guillotine door of the tunnel was opened, and the male had the opportunity to explore the female's outdoor enclosure. In the third period C (June 17, 2015–June 24, 2015), both individuals spent the daytime (8 a.m.–6 p.m.) in their respective outdoor enclosures and the nighttime in their indoor enclosures. During this period, the two individuals were both kept in their outdoor enclosures and were able to see each other. During the fourth period D (June 29, 2015–July 6, 2015), the management of the two subjects was similar to the first period, with the male in the outdoor enclosure during the day, from 8 a.m. to 6 p.m., and the female staying in the indoor enclosure. In this period, data collection on the female was carried out through video recordings of a fixed camera in her indoor enclosure, to assess behavioral differences between the indoor and outdoor enclosures. During the fifth period E (July 8, 2015–July 13, 2015), the morning hours followed the same schedule as the fourth period, while in the afternoon, the zoo adopted the summer schedule, and the male's enclosure was closed later in the evening.

**Table 1:** Hours of usage of outdoor and indoor areas by each animal and of observations during different research periods (morning and afternoon).

Period^1^	Subj^2^	Outdoor	Indoor	Morning observations	Afternoon observations
A	M	9:00 a.m.–6:00 p.m.	6:00 p.m.–9:00 a.m.	9:30 a.m.–10:30 a.m.	3:00 p.m.–4:00 p.m.
	F	6:00 p.m.–9:00 a.m.	9:00 a.m.–6:00 p.m.	8:00 a.m.–9:00 a.m.	6:00 p.m.–7:00 p.m.
B	M	9:00 a.m.–6:00 p.m.*	6:00 p.m.–9:00 a.m.	9:30 a.m.–10:30 a.m.	3:00 p.m.–4:00 p.m.
	F	6:00 p.m.–9:00 a.m.	9:00 a.m.–6:00 p.m.	8:00 a.m.–9:00 a.m.	6:00 p.m.–7:00 p.m.
C	M	8:00 a.m.–6:00 p.m.	6:00 p.m.–8:00 a.m.	8:00 a.m.–10:00 a.m.	3:00 p.m.–5:00 p.m.
	F	8:00 a.m.–6:00 p.m.	6:00 p.m.–8:00 a.m.	8:00 a.m.–10:00 a.m.	3:00 p.m.–5:00 p.m.
D	M	8:00 a.m.–6:00 p.m.	6:00 p.m.–8:00 a.m.	8:00 a.m.–9:00 a.m.	3:00 p.m.–4:00 p.m.
	F	6:00 p.m.–8:00 a.m.	8:00 a.m.–6:00 p.m.	9:00 a.m.–10:00 a.m.**	4:00 p.m.–5:00 p.m.**
E	M	8:00 a.m.–8:30 p.m.	8:30 p.m.–8:00 a.m.	8:00 a.m.–9:00 a.m.	7:00 p.m.–8:00 p.m.
	F	8:30 p.m.–8:00 a.m.	8:00 a.m.–8:30 p.m.	7:00 a.m.–8:00 a.m.	8:00 p.m.–9:00 p.m.

*Note:* A, from May 27, 2015, to June 5, 2015; B, from June 8, 2015, to June 16, 2015; C, from June 17, 2015, to June 24, 2015; D, from June 29, 2015, to July 6, 2015; E, from July 8, 2015, to July 13, 2015. The observations were conducted when the animals were in the outdoor areas, considering the management of the animals according to the familiarization program designed and also following the routine of the zoo.

Abbreviations: F, female; M, male; Subj, subjects.

* Access to the female's outdoor (from 3.00 p.m. to 4.00 p.m.).

** Indoor observations.

For each period and each individual, two 60‐min data collection sessions were conducted daily, one in the morning and one in the afternoon, totalizing 12 data collection sessions for each research period and each subject. In total, 120 recording sessions were conducted for both individuals, amounting to 120 h of observation (Table 1). Focal animal sampling (Altmann 1974) was carried out with a stopwatch and a paper ballot when the animals were in their outdoor enclosures, except for period D, during which the female was observed in her indoor area through a fixed camera. This collection method was performed to evaluate the duration of different behaviors for each animal. The date of the session, subject, start time for each observation, the start of each action, the area of the enclosure where the subject was, and any comments were recorded on the paper ballot. If observed categories were mutually exclusive, the onset of the following action indicated the end of the previous behavior (Altmann 1974).

### 2.3 Fecal Metabolite Hormone Determination

Seventy‐seven fecal samples (*n* = 40 for the male, *n* = 37 for the female) were processed for the determination of steroid hormone metabolites concentrations. Samples were collected in the morning of the B, C, D, and E periods. During period A, it was not possible to collect samples due to technical issues. All samples were immediately identified and stored in PPL bags at −20°C, until extraction and assay.

Extraction of steroid hormone metabolites from feces was performed as described by Schatz and Palme (2001). Five milliliters of a methanol: water (v/v 4:1) solution was added to 500 mg (wet weight) of feces in capped glass tube vials. Afterward, vials were vortexed for 30 min using a multitube pulsing vortexer. Following centrifugation (1500*g* for 15 min), 5 mL ethyl ether (BDH Italia, MI, Italy) and 0.2 mL NaHCO_3_ (5%) (Sigma Chemical Co., St. Louis, MO, USA) were added to 1 mL supernatant. This solution was vortexed for 1 min on multitube pulsing vortexer and centrifuged for 5 min (1500 g). The ether portion was then separated by sucking it with a pipet and evaporated under an air‐stream suction hood at 37°C. Dry residue was finally redissolved into 0.5 mL PBS 0.05 M, pH 7.5.

Concentrations of fecal cortisol (FCM), testosterone (FTM), and progesterone (FPM) metabolites were determined using RIA (Radio Immune Assay) based on binding of 3H‐steroid by competitive adsorption (Fenske and Schönheiter 1991). A recovery test on five replicates was performed by adding 125, 250, 500, or 1000 pg of 3H‐cortisol, testosterone, and progesterone to 500 mg of feces and incubating for 30 min at room temperature. The extraction was performed as described above, yielding a mean percentage recovery of 88.78% ± 2.34. The assay was carried out according to Tamanini et al. (1983) for FCM, Gaiani et al. (1984) for FTM, and Seren, Leopold, and Bolelli (1974) for FPM and analysis was performed in duplicate. One hundred microliters of 3H‐cortisol, testosterone, or progesterone and 100 μL of an anti‐hormones antibody (dilution 1:20,000) were added to 100 μL of the solution obtained from the extraction procedure. After incubation at +4°C for 18 h, the free steroid was separated from the bound by the addition of 1 mL of a solution of charcoal 1% (Sigma Chemical Co.) + 0.025% dextran (Sigma Chemical Co.), and incubation at +4°C for 15 min followed by centrifugation (4000*g*) for 4 min at +4°C. The supernatant containing the hormone bound to its antibody was then decanted into scintillation vials and measured in a liquid scintillation β counter (Perkin–Elmer Life Science Inc.).

In‐house validation of the analysis revealed the following parameters: cortisol, sensitivity 3.10 pg/100 μL, intra‐assay variability 6.8%, interassay variability 9.3% for cortisol; Testosterone, sensitivity 2.80 pg/100 μL; intra‐assay variability 5.7%; and interassay variability 10.1%; Progesterone, sensitivity 2.72 pg/100 μL; intra‐assay variability 9.28%; interassay variability 13.95%.

Radioactivity was determined using a liquid scintillation β counter and a linear standard curve (ad hoc designed software program: Motta and Degli Esposti 1981). All concentrations were expressed in pg/mg of fecal.

To determine the parallelism between hormones standards (Sigma Chemical Co.) and endogenous hormones, fecal samples from the two animals were serially diluted with PBS 0.05 M, pH 7.5 to obtain volumes of 50, 25, 10, and 5 ll. Parallelism was assessed between these serial dilutions and standards (ranging from 7.81 to 1000 pg/100 ll tube vial, prepared in buffer). A nonlinear regression test was used to assess parallelism between standard and endogenous hormones, and a high degree of parallelism (*p* < 0.01) was observed between the standard and the diluted samples' curves.

Intestinal transit was assessed by administering grains and corn with the meal to the animals, and then detecting the undigested residues in the feces. A transit time of 16–18 h was estimated.

### 2.4 Statistical Analysis

The statistical analysis was conducted using RStudio (version 2023.06.0 + 421), an integrated environment for R that facilitates statistical programming and data visualization (Posit team 2023). To explore behavioral differences among individuals during the designated periods, the normality of distribution was assessed using the Shapiro–Wilk test, and due to the non‐normal distribution of data, we employed the Friedman test to compare the duration of behavioral categories listed in the ethogram, and the Nemenyi test was performed as a post hoc analysis.

To investigate the relationship between hormonal metabolites levels and the sampling period, we utilized the “lme4” R package (Bates et al. 2015) to run a Generalized Linear Model (GLM). FTM concentrations of the male and FCM levels for both subjects were set as outcome variables in the respective GLMs, while the period was considered a fixed variable. Before running the analysis, the outcome variables underwent a logarithmic transformation. The normality of residuals was assessed visually using histograms and statistically using the Shapiro–Wilk test. In addition, the independence of residuals and homoscedasticity were tested respectively through Durbin–Watson and Breusch–Pagan tests.

All results have been reported as a median and interquartile range, and statistical significance was considered for *p* values less than 0.05.

## 3 Results

### 3.1 Behavioral Analysis

According to the statistical analysis, there were no significant changes in the duration of not visible (female: *χ* ^2^ = 8.46, d.f. = 4, *p* = 0.076; male: *χ* ^2^ = 1.63, d.f. = 4, *p* = 0.804) and maintenance behaviors (female: *χ* ^2^ = 4.24, d.f.= 4, *p* = 0.374; male: *χ* ^2^ = 6.53, d.f. = 4, *p* = 0.163) among the five periods considered for both subjects.

Regarding the male subject, the Friedman test showed a significant difference for abnormal behaviors throughout all periods (*χ* ^2^ = 18.5, d.f. = 4, *p* = 0.001, Kendall's *W* = 0.386). The post hoc analysis revealed a significantly longer duration of abnormal behaviors during period C compared to period A (*p* = 0.0035). Furthermore, the Friedman test showed a significant difference for duration of locomotion throughout all periods (*χ* ^2^ = 15.2, d.f. = 4, *p* = 0.004, Kendall's *W* = 0.316). Considering the post hoc analysis, the duration of locomotion was significantly longer during period C compared to periods A (*p* = 0.0167) and E (*p* = 0.0035). On the other hand, the duration of seeking behavior, which resulted in significantly different among all the periods (*χ* ^2^ = 27.7, d.f. = 4, *p* = 0.00001, Kendall's *W* = 0.576), was significantly shorter during periods C and E compared to periods A and B (C vs. A, *p* = 0.0167; C vs. B, *p* = 0.0006; E vs. A, *p* = 0.0017; E vs. B, *p* = 0.0364), as during period D compared to period A (*p* = 0.0028). Lastly, the Friedman test showed that the duration of marking behaviors was significantly different throughout all periods (*χ* ^2^ = 13.5, d.f. = 4, *p* = 0.01, Kendall's *W* = 0.282). The post hoc analysis revealed a significantly shorter duration of marking behavior in periods A and D compared to period B (A vs. B, *p* = 0.025; D vs. B, *p* = 0.011 (Table 2).

**Table 2:** The median (25°–75° quantiles) duration in seconds of male behavioral categories during the five considered periods.

	A	B	C	D	E
Ab	0 (0–0)^b^	0 (0–156.0)^a,b^	1450.0 (1244.75–2022.5)^a^	552.0 (0–1182.75)^a,b^	270.0 (0–2153.75)^a,b^
L	257.5 (163.5–390.5)^b^	310 (246.25–438.25)^a,b^	692.5 (431.5–811.75)^a^	349.5 (235.75–446.25)^a,b^	208.0 (162.0–378.0)^b^
Main	1709.0 (1207.75–2042.5)^a^	877.0 (410.0–2378.5)^a^	638.0 (161.5–990.75)^a^	1621.5 (950.5–2589.25)^a^	885.5 (81.25–2587.25)^a^
Mar	18.0 (9.0–85.25)^b^	160.5 (106.25–283.25)^a^	38.0 (16.25–90.25)^a,b^	26.5 (14.0–54.0)^b^	75.5 (23.25–134.0)^a,b^
Nv	0 (0–102.0)^a^	32.0 (20.75–175.25)^a^	26.0 (0–77.0)^a^	15.5 (0–50.75)^a^	57.0 (0–119.25)^a^
S	1212.5 (1163.5–1777.0)^a^	1069.5 (661.25–1827.75)^a,b^	452.0 (269.25–563.75)^c^	393.0 (297.75–580.25)^b,c^	421.0 (333.75–484.0)^c^

*Note:* A, from May 27, 2015, to June 5, 2015; B, from June 8, 2015, to June 16, 2015; C, from June 17, 2015, to June 24, 2015; D, from June 29, 2015, to July 6, 2015; E, from July 8, 2015, to July 13, 2015.

Abbreviations: Ab, abnormal behavior; L, locomotion; Main, maintenance; Mar, marking; Nv, not visible; S, seeking.

^a,b,c^Values within rows with different superscripts differ for *p* < 0.05 at the Nemenyi test.

Considering the female subject, the Friedman test showed a significant difference for abnormal behavior throughout all periods (*χ* ^2^ = 18.8, d.f. = 4, *p* = 0.001, Kendall's *W* = 0.392). The post hoc analysis revealed that, during period D, she showed a significantly shorter duration of abnormal behavior compared to periods B (*p* = 0.001), C (*p* = 0.0302), and E (*p* = 0.036). Additionally, considering the Friedman test, also the duration of marking behavior resulted significantly different among the five periods (*χ* ^2^ = 16.4, d.f. = 4, *p* = 0.002, Kendall's *W* = 0.343). The post hoc analysis showed that in period D, the duration of marking behavior was shorter compared to periods A (*p* = 0.0035), B (*p* = 0.0302), and C (*p* = 0.0249). In the meantime, the results of the Friedman test showed that also locomotion duration was significantly different throughout all periods (*χ* ^2^ = 26.5, d.f. = 4, *p* = 0.00001, Kendall's *W* = 0.551). In particular, the post hoc analysis revealed a significant shorter duration of locomotion in period D compared to period A (*p* = 0.0364), B (*p* = 0.011), C (*p* = 0.0006) and E (*p* = 1.8^−05^). Finally, considering Friedman test, the duration of seeking behavior significantly differed among the five periods during periods (*χ* ^2^ = 14.3, d.f. = 4, *p* = 0.006, Kendall's *W* = 0.299). Specifically, the post hoc analysis revealed that, during periods A and D, the females recorded a significantly shorter duration of seeking behavior compared to period E (E vs. A, *p* = 0.025; E vs. D, *p* = 0.017; Table 3).

**Table 3:** The median (25°–75° quantiles) duration in seconds of female behavioral categories during the five considered periods.

	A	B	C	D	E
Ab	1360.0 (0–2997.25)^a,b^	2521.0 (975.75–2960.5)^a^	1863.0 (1231.5–2336.75)^a^	0 (0–0)^b^	1955.0 (1209.5–2376.5)^a^
L	160.0 (120.5–298.0)^a^	235.5 (156.0–428.5)^a^	369.0 (230.5–483.25)^a^	0 (0–15.0)^b^	476.5 (353.0–519.0)^a^
Man	23.5 (6.75–763.25)^a^	115.0 (1.5–468.75)^a^	249.5 (55.25–647.75)^a^	2287.5 (0–3192.0)^a^	25.5 (2.25–851.0)^a^
Mar	42.5 (2.25–60.5)^a^	24.0 (0–43.5)^a^	21.0 (0–40.75)^a^	0 (0–0)^b^	13.5 (11.0–22.0)^a,b^
Nv	0 (0–1021.75)^a^	7.0 (0–57.0)^a^	0 (0–35.5)^a^	103.5 (0–3600.0)^a^	0 (0–0)^a^
S	354.5 (261.25–512.25)^b^	443.0 (332.25–1021.0)^a,b^	608.0 (432.75–791.75)^a,b^	169.5 (0–458.25)^b^	736.5 (555.25–922.25)^a^

*Note:* A, from May 27, 2015, to June 5, 2015; B, from June 8, 2015, to June 16, 2015; C, from June 17, 2015, to June 24, 2015; D, from June 29, 2015, to July 6, 2015; E, from July 8, 2015, to July 13, 2015.

Abbreviations: Ab, abnormal behavior; L, locomotion; Main, maintenance; Mar, marking; Nv, not visible; S, seeking.

^a,b,c^ Values within rows with different superscripts differ for *p* < 0.05 at the Nemenyi test.

### 3.2 Endocrinological Results

**Figure 1:**
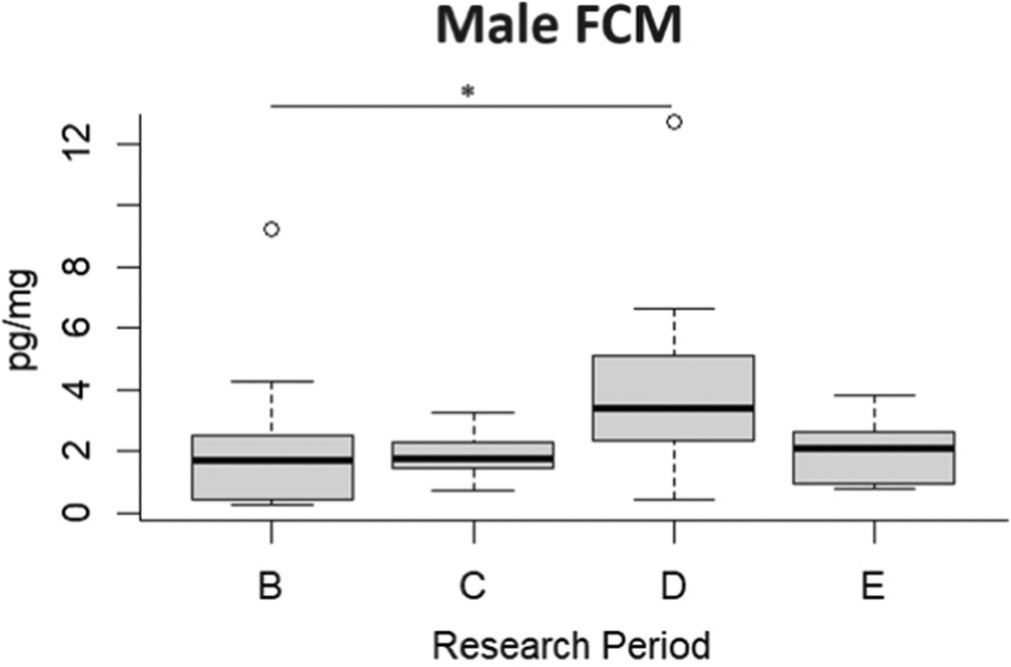
Fecal cortisol metabolite level (pg/mg) of the male subject in the four different research periods (B, C, D, E). The bar within the box represents the median, the borders of the box are upper quartiles, the bottom, and top whiskers signify the lowest and highest cases within 1.5 times interquartile range, and outliers are shown through white circles.*Significant GLM results (*p* < 0.05).

Considering FCM concentration, in the male fossa, it was significantly higher in period D compared to period B (*β* ± SE = 0.906 ± 0.406, *t*‐value = 2.230, *p* = 0.033) (Figure 1).

**Figure 2:**
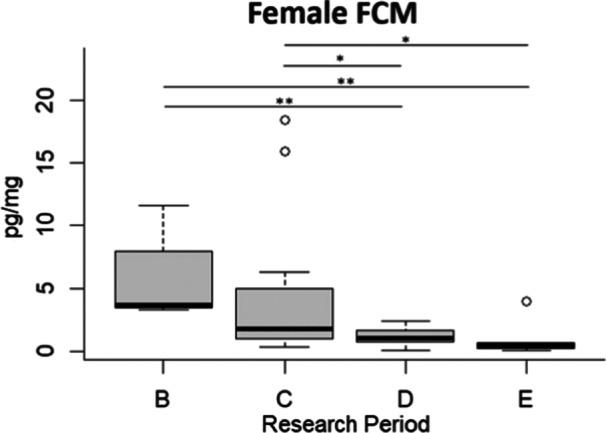
Fecal cortisol metabolite levels (pg/mg) of the female subject in the four different research periods (B, C, D, E). The bar within the box represents the median, the borders of the box are upper quartiles, the bottom, and top whiskers signify the lowest and highest cases within 1.5 times interquartile range, and outliers are shown through white circles.*Significant GLM results (*p* < 0.05); **Significant GLM results (*p* < 0.01).

On the contrary, the FCM concentration of the female subject was significantly higher during period B compared to periods D (*β* ± SE = −1.776 ± 0.542, *t*‐value = −3.275, *p* = 0.003) and E (*β* ± SE = −2.298 ± 0.630, *t*‐value = −3.646, *p* = 0.001), and significantly higher in period C compared to period D (*β* ± SE = −0.995 ± 0.484, *t*‐value = −2.058, *p* = 0.049) and E (*β* ± SE = −1.517 ± 0.580, *t*‐value = −2.613, *p* = 0.014). However, we did not record significant differences between periods B and C (*β* ± SE = −0.7811 ± 0.406, *t*‐value = 2.230, *p* = 0.1445), and between periods D and E (*β* ± SE = −0.522 ± 0.600, *t*‐value = −0.869, *p* = 0.392; Figure 2).

**Figure 3:**
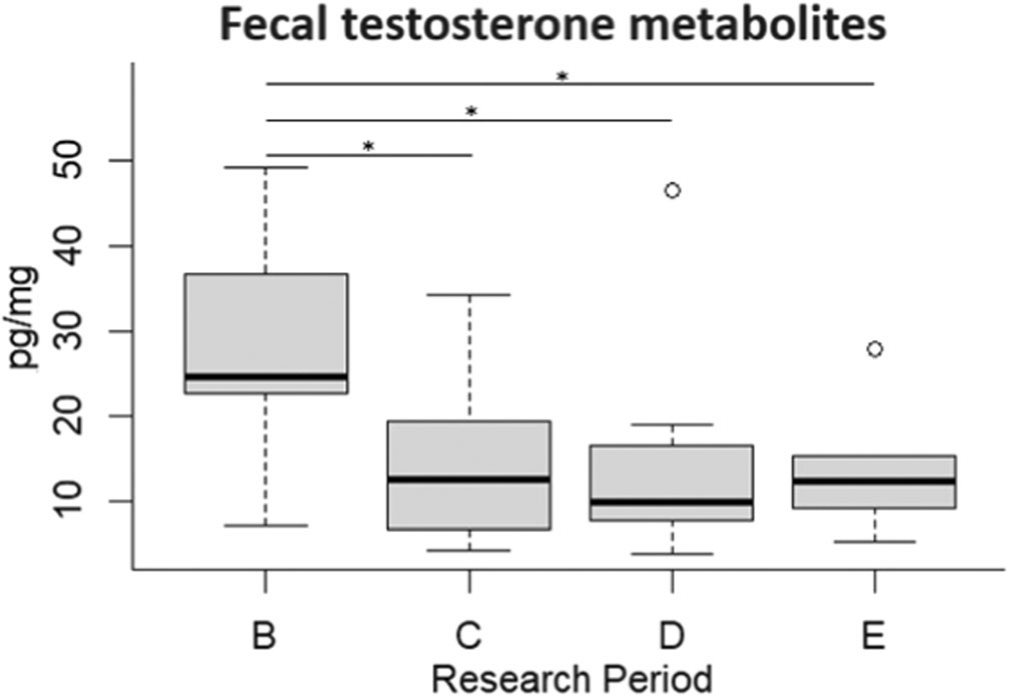
Fecal testosterone metabolite levels (pg/mg) of the male subject in the four different research periods (B, C, D, E). The bar within the box represents the median, the borders of the box are upper quartiles, the bottom, and top whiskers signify the lowest and highest cases within 1.5 times interquartile range, and outliers are shown through white circles. *Significant GLM results (*p* < 0.05).

Regarding sexual hormones, male FTM concentration was significantly higher in period B compared to period C (*β* ± SE = −0.716 ± 0.292, *t*‐value = −2.450, *p* = 0.020), period D (*β* ± SE = −0.755 ± 0.306, *t*‐value = −2.464, *p* = 0.019), and period E (*β* ± SE = −0.707 ± 0.343, *t*‐value = −2.063, *p* = 0.047; Figure 3).

**Figure 4:**
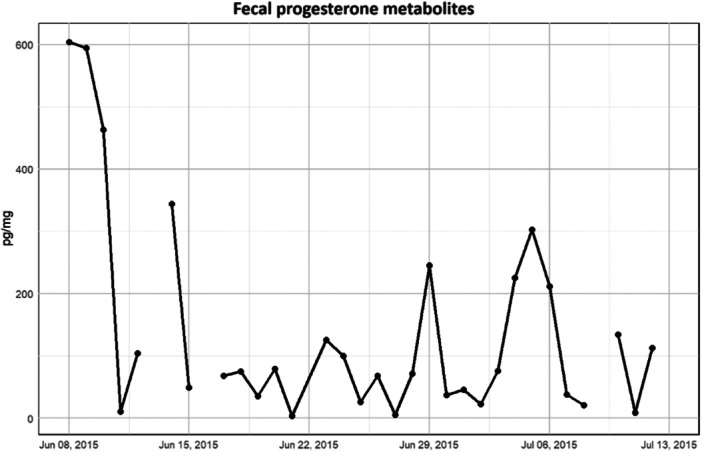
Daily trend of fecal progesterone metabolites (FPM) concentration (pg/mg).

Regarding the FPM, considering the physiologically fluctuations of this hormone depending on the estrous cycle (Poli et al. 2018), rather than comparing fecal levels across different periods, we chose to utilize descriptive statistics to outline its trend. As depicted in Figure 4, the concentration of FPM exhibited a range from a minimum of 4.09 pg/mg to a peak of 604.39 pg/mg. Notably, the highest FPM concentration within the data set was observed during the first 3 days of period B, spanning from June 8, 2015, to June 16, 2015.

## 4 Discussion

Because of their elusive nature, conducting studies on the reproductive and behavioral aspects of the fossa under human care can offer invaluable insights into this enigmatic species and make significant contributions to its conservation efforts. Therefore, our research aimed to describe behavioral patterns and hormonal fluctuations of a couple of fossa hosted in a zoo.

The two subjects of the study recorded fluctuations in their behaviors across different research periods. Considering abnormal behavior, both individuals exhibited pacing, which is a repetitive locomotor stereotypy commonly seen in carnivores. This behavior involves the subjects engaging in repetitive walking patterns that often resemble the shape of an “8,” a circular path, or a straight line (Carlstead 1998). Pacing in fossa has been documented as both stereotypies and anticipatory behaviors. An anticipatory behavior is shown by an animal in the period between the signaling of an event and its actual occurrence and is more pronounced when routines in management practices are highly predictable (Harley, O'Hara, and Rose 2021; Ward, Sherwen, and Clark 2018). However, as the behavior did not coincide with any predictable event (e.g., feeding time, the arrival of keepers, or enclosure entry), we were more inclined to classify it as stereotypic behavior in both subjects of our study. Stereotypies, or repetitive and unchanging actions without an apparent purpose, tend to emerge due to motivational frustration and boredom (Mason and Latham 2004). In a review focused on the husbandry protocols and enclosure conditions for fossa worldwide, Harley, O'Hara, and Rose (2021) highlight that a significant proportion of fossa exhibit abnormal behaviors, with pacing being the most commonly recorded. Indeed, various studies have demonstrated that species with expansive ranges of activity, regardless of their body size, are more prone to develop stereotypical behaviors under controlled environments (Clubb and Mason 2007; Kroshko et al. 2016). Furthermore, stereotypies may develop into habits that are difficult to address or correct. These habits may not be directly linked to the current housing conditions but may instead be remnants of previous suboptimal situations (Mason and Latham 2004). In the meantime, it should be considered that stereotypies can have a coping function and preventing them can increase arousal (Cooper and Nicol 1993; McGreevy and Nicol 1998; McBride and Cuddeford 2001). However, evidence suggests that reducing stereotypies can enhance welfare conditions, and the goal of management should be to mitigate the stereotypies before their expression (Harley, O'Hara, and Rose 2021). The male individual in our study, as specified in the Materials and Methods section, exhibited pacing behavior upon his arrival at the zoo, which was partially addressed with a targeted enrichment program. He did not show the stereotypy during research period A, but its expression began to appear in research period B and peaked during period C when both subjects were present in their respective outdoor enclosures. Differently, for the female, we never recorded pacing during research period D, when we observed her in her indoor area, but she displayed it during the other research periods, with a peak in period B. During this period, the male was allowed to enter the female's outdoor enclosure for 1 h when she was not present. We could hypothesize that the presence of a conspecific in the nearby outdoor area or the scent of other individuals within their own territory could potentially serve as sources of frustration and stress. In particular, the female, recorded significantly higher levels of FCM during research periods B and C in comparison to periods D and E. In many zoos and breeding facilities, animals are often housed in close proximity to conspecifics, creating a “sense but can't interact” environment (Potratz et al. 2023). This practice can lead to stress and trigger abnormal behaviors (Clubb and Mason 2007; Morgan and Tromborg 2007). In particular, fossa are solitary animals, and the presence of conspecifics and other species may lead to the display of abnormal behaviors. In their review, Harley, O'Hara, and Rose (2021) underline how pacing can become more pronounced when fossa of the opposite sex are housed in the olfactory, auditory, or visual range of a conspecific, probably because of overstimulation or driven by the frustration of moving away or approaching the other individual.

However, it is important to note that sensory awareness, even without direct interaction, has been acknowledged as a form of enrichment that can enhance activity and overall well‐being in animals under human care (Wells 2009). Indeed, during research period B, the male showed a higher duration of seeking behavior compared to periods C and E, and also increased marking behaviors significantly. In addition, in period B his FCM concentration was significantly lower compared to period D, when the male was not allowed to access the outdoor area of the female and the female remained inside when he was outside. For carnivores under human care, sensory cues, particularly olfactory stimuli, have been shown to enhance exploratory behavior and activity levels in species such as African wild dogs (*Lycaon pictus*; Rafacz and Santymire 2014) or black‐footed cats (*Felis nigripes*; Wells and Egli 2004). Olfaction, being the dominant sense for many mammals, provides essential sensory sampling and exploration opportunities (Marneweck, Jürgens, and Shrader 2018). The behaviors of “marking” and “sniffing” enable these animals to exchange vital information regarding individual identification, the presence of potential mates or threats, and territorial boundaries. By means of pheromones or chemical signals released through feces, urine, or exocrine glands, these behaviors facilitate interactions among individuals of the same species, conveying information about different aspects such as nutrition, social relationships, and reproductive conditions (Dickie 2005; Dehnhard 2011). Odor‐guided behaviors can play crucial roles in mate selection, sexual behavior, foraging, and territoriality, especially in solitary species, like fossa (Dickie 2005; Wyza et al. 2020). We might suppose that the heightened marking and seeking behaviors exhibited by the male could have been triggered by both the opportunity to enter and explore the female's territory, as well as their reproductive status. In fact, taking into account the steroid hormone pattern of the female, the peak in FPM level in the initial days of research period B could suggest a potential former ovulation. This could also explain the higher FTM concentration in the male during period B. While a more extended monitoring period of fecal steroid hormones' metabolites would have provided us with more comprehensive information, existing literature supports this hypothesis (Dickie 2005; Vogler et al. 2009). Indeed, fossa are solitary monoestrous animals whose breeding season in the northern hemisphere takes place from March to July, and they employ marking behaviors to indicate their receptiveness to mating and to synchronize their physiological state (Dickie 2005; Vogler et al. 2009). The median marking behavior of the female was higher in period A, even if not significantly compared to periods B, C, and E, while we did not record any marking in period D.

It is important to note that during period D, our observations were conducted while the female was situated within her indoor enclosure. Throughout this observation period, the duration of locomotion, marking, and seeking behaviors were lower compared to the other research periods. Notably, the female exhibited primarily maintenance behaviors while indoors. The decrease in diversity of activities could potentially have been because the indoor enclosure is smaller and less enriched than the outdoor habitat. It should be considered that fossa has a complex ecology. In their natural habitat, fossa inhabits dense forests and occupy expansive territories, covering multiple kilometers daily. They are highly active both during the day and night, especially just before and after dawn and dusk, and they possess the ability to hunt effectively in both the forest canopy and on the ground (Winkler 2002). Wild fossa dedicates around 70% of their time to patrolling their territory, hunting for food, or seeking a mate. During the remaining time, typically the hottest daytime and coldest nighttime segments, they rest either on the ground or on larger branches. When covering longer distances, fossa generally favor traveling on the ground. Despite their substantial time spent at ground level, they regularly climb trees, particularly when marking their territory, hunting, and mating. They exhibit anatomical adaptation to climbing, such as their long tail for stability or their sharp claws (Winkler 2002). Therefore, to enhance the welfare of fossas, it is recommended to increase the frequency of species‐specific individualized environmental enrichment and the complexity of the environment. This could include establishing an environment that offers abundant climbing options, a variety of ground surfaces, vegetation, and extensive chances for exploration and enrichment. This strategy can help to counteract the propensity of this species to develop repetitive behaviors (AZA Small Carnivore TAG 2011; Harley, O'Hara, and Rose 2021). Nevertheless, it is important to note that during period D, no repetitive or abnormal behaviors were documented. We cannot exclude that the absence of olfactory or auditory stimuli from the male conspecific could have contributed to a decrease in the female's stress levels. Indeed, it is likely that in the indoor environment, being more secluded, the female felt in her own territory and experienced less stress and greater relaxation. At the same time, we cannot exclude that also being completely hidden from visitors might have played a role in this outcome. However, the lack of data about the actual number and potential disturbance of visitors represents a limitation of our study, and we cannot confirm this hypothesis. The impacts of visitors on zoo animals have been extensively discussed in the literature, with different results and individual variability. Although the presence and proximity of visitors may occasionally lead to stress in animals, resulting in the display of abnormal behaviors (Fernandez et al. 2009; Boyle et al. 2020; Harley, O'Hara, and Rose 2021), further research in this area is necessary to comprehensively understand the effects of visitors on fossa behavior.

## 5 Conclusion

The noninvasive study of fossa behavior and endocrinological status under human care offers valuable insights into the challenges and strategies involved in managing and conserving endangered species under human care. The observed fluctuations in behavior and hormonal levels underline the complexities of replicating natural conditions in a controlled environment. It is important to recognize that addressing welfare and reproduction issues needs a comprehensive understanding of the animals' natural ecology, and, in this solitary species with a wide home‐range, the management of the socialization process can be seriously challenging. Social and environmental factors, including the presence of conspecifics and the availability of sensory cues, play pivotal roles in influencing behavior. Creating enriched environments that stimulate natural behaviors while accounting for species‐specific requirements is a necessity, and future research is needed to delve into the endocrinological and behavioral aspects of fossa under human care, such as the study of the visitors' influence on this species.

In conclusion, even if this study focused on only two individuals and our results cannot be generalized, it contributes to evidence of some of the possible challenges faced in maintaining the welfare of fossa under human care. By acknowledging the intricate interplay of social, environmental, and reproductive factors and by drawing from insights into their natural behaviors, strides can be made in enhancing the welfare of these animals and contributing to their conservation efforts.

## Author Contributions

**Giovanna Marliani:** data curation, writing–original draft. **Giovanni Buonaiuto:** writing–original draft, writing–review and editing. **Pier Attilio Accorsi:** writing–review and editing, project administration. **Silvia Calamandrei:** investigation, observer. **Camillo Sandri:** investigation, writing–original draft. **Caterina Spiezio:** conceptualization, supervision, methodology.

## Ethics Statement

The experimental procedures are only observational and noninvasive and comply with the ethical standards on animal experimentation in accordance with EU Directive 2010/63/EU for animal experiments.

## Conflicts of Interest

The authors declare no conflicts of interest.

## Supporting information


**Table S1.** Ethogram employed in the study.

## Data Availability

The data that support the findings of this study are available from the authors upon request.
