# Supplementary material for: Investigating the Welfare of Zoo‐Housed Cryptoprocta ferox: Behavioral Observations and Hormonal Profiling
Source: Zoo Biol. 2024 Dec 19;44(2):178–88. doi: 10.1002/zoo.21884 (PMC11963218; doi:10.1002/zoo.21884)
Supplement: Supplementary file 1 — Table S1. Ethogram employed in the study. [file ZOO-44-178-s001.docx]

| ***Class*** | ***Behavioural category*** | ***Definition*** |
| --- | --- | --- |
| Other activities | Interaction with Enrichment | Looking for food given as enrichment or manipulation of objects |
|  | Auto-grooming | Licking or scratching a part of the body |
|  | Maintenance | Eating, drinking, defecation, urination, sleep, yawn, stretch |
| Marking | Marking trees and ground | Rubbing body part, head or hindquarters against tree or soil |
| Locomotion | Locomotion to the ground | The subject walks, runs and moves on the ground |
|  | Arboreal Locomotion | The subject walks, runs and moves on the trees |
| Exploratory | Attention | The subject stands or sits in an alert state |
|  | Explorative behaviour | The subject moves and looks around |
|  | Sniffing | The subject sniffs the air, the ground and objects |
| Individual pathological behaviour | Pacing | The subject repeatedly follows an eight-shaped path without changing its behaviour and in an aphylactic manner |
|  | Immobilism | The subject stand still and rigid, staring at a fixed point |
| Not observed | Not observed | The subject is not visible to the observer |
